# Ameliorative effect of vitamin E on trichloroethylene-induced nephrotoxicity in rats

**DOI:** 10.15171/jnp.2017.29

**Published:** 2016-12-20

**Authors:** Mojgan Heydari, Massumeh Ahmadizadeh, Kambiz Ahmadi Angali

**Affiliations:** ^1^Department of Occupational Health, Engineering, School of Health, Ahvaz Jundishapur University of Medical Sciences, Ahvaz, Iran; ^2^Physiology Research Center, Ahvaz Jundishapur University of Medical Sciences, Ahvaz, Iran; ^3^Department of Statistics and Epidemiology, School of Health, Ahvaz Jundishapur University of Medical Sciences, Ahvaz, Iran

**Keywords:** Vitamin E, Trichloroethylene, Malondialdehyde, Glutathione

## Abstract

**Background:**

1,1,2-Trichloroethylene (TCE) is an important organic solvent which is widespread in the environment. Work place exposure to TCE has been associated adverse effects in many organs including kidney. Vitamin E is an antioxidant that can overcome oxidative stress.

**Objectives:**

The aim of the present study is to examine the role of vitamin E against destructive effects of TCE on rat kidney.

**Materials and Methods:**

A total of 35 male Wistar rats were randomly divided into seven groups of equal number in each. The rats in group I were the controls received vehicle only. Animals in groups III, V and VII received intraperitoneal injection (i.p) of corn oil. Rats in groups of II, IV, and VI were received vitamin E at a dose of 200 mg/kg; 30 minutes later, animals were received TCE (i.p) at doses of 1000 mg/kg (groups II and III), 1500 mg/kg (groups of IV and V), and 2000 mg/kg (groups of VI and VII) respectively. The experiment repeated for 7 consecutive days. Twenty-four hours after last administration, animals were killed with overdose of sodium pentobarbital. Blood samples were analyzed for blood urea nitrogen (BUN) and creatinine (Cr). One part of the kidney tissues were excised for measuring malondialdehyde (MDA) and glutathione (GSH) concentrations. Another part were excised for histopathological estimation.

**Results:**

TCE induced a dose-dependent elevation in BUN, Cr, MDA and markedly decreased GSH level when compared to those in control rats. TCE-induced dose-dependent injury in rat kidney tissue. Vitamin E significantly decreased BUN, Cr, MDA and increased GSH levels and protected kidney damage in TCE treated animals.

**Conclusions:**

The observations suggest that vitamin E may have a protective effect against TCE-induced oxidative stress in the rat kidney.

**Implication for health policy/practice/research/medical education:**

In an experimental study, we found that vitamin E as an antioxidant agent protects kidney against 1,1,2-trichloroethylene (TCE) induced nephrotoxicity. The mechanism of this renoprotective effects mainly includes amelioration of lipid peroxidation produced by TCE as well as elevation of glutathione (GSH).

## 1. Background

Trichloroethylene (TCE) is a volatile, colorless, inflammable organic solvent with sweet smell like that of chloroform. TCE is widely used as one of the most common organic solvents in petroleum extraction industries, laundries, degreasing and cleaning of metal components (1,2). This substance affects directly people occupationally exposed to it and indirectly, through contamination of the environment, people using surface water sources (1,2). Since kidneys are the main site for the excretion of metabolic waste, they are constantly exposed to toxins. Any kind of kidney damage may disturb metabolism of the body. Pathological studies on the kidney show that TCE and its metabolites, including dichlorovinyl glutathione and dichlorovinyl cysteine, cause damage to renal tubules, mainly, damage to renal proximal tubules cells (4-8). Exposure to TCE substantially increases creatinine (Cr) and blood urea nitrogen (BUN) that are indicative of nephrotoxicity caused by TCE (9,10). Nephrotoxicity caused by TCE is associated with its reactive metabolites derived from incorporation of TCE in glutathione (GSH) conjugation reaction which occurs in the kidney (5,9,10). Renal effects of TCE have generally been associated to GSH conjugation pathway and its subsequent metabolism. Ultimately, TCE and its metabolites are mainly excreted via urine (5-10).

Increasing evidences indicated that TCE induced oxidative stress to rat kidney tissue and alter their metabolic functions (5,8,10-14). Numerous studies have shown that antioxidant agents can protect kidney against TCE-induced toxicity (10,12-14). Siddiqi et al reported that pretreatment with hesperidin as an antioxidant agent considerably reduced lipid peroxidation, elevation of oxidative enzymes level, blood urea and Cr concentrations in rats exposed to TCE. These results indicate that hesperidin can function as a protective agent against TCE caused nephrotoxicity (12).

Vitamin E (α-tocopherol) is the most important chain breaking antioxidant in the body. Studies indicated that vitamin E inhibits the production of reactive oxygen species and lipid peroxyl radicals and prevents peroxidation of unsaturated fatty acids. It is a non-enzymatic antioxidant that can overcome oxidative stress. Vitamin E is an important lipid-soluble antioxidant mostly abundant in cell membrane, and helps to maintain membrane stability (15). Zhu et al showed that vitamin E could effectively prevent cytotoxicity caused by TCE in human skin keratinocytes through inhibition of superoxide and increasing activity of antioxidant enzymes (16).To our knowledge the effects of vitamin E on TCE produced nephrotoxicity has not been reported previously.

## 2. Objectives

The aim of the present study was to investigate the effects of vitamin E on TCE-induced nephrotoxicity.

## 3. Materials and Methods

### 3.1. Chemicals

TCE and corn oil were prepared from Merck (Germany) and Vitamin E from SERVA. Other chemicals and reagents used were from Merck and Sigma-Aldrich.

### 3.2. Animals

Adult male Wistar rats weighing 180-200 g were provided from the center of laboratory animal husbandry of Jundishapur University of Ahvaz, Iran. Rats were kept in experimental laboratory for one week before the start of the experiment as adaptation period. Animals were maintained in special cages placed in a room with proper ventilation (23^°^C) with equal light and dark cycle of 12 hours a day throughout the experiment. Rats were free access to tap water and food formulated especial for them. The standard pellet rat diet and tap water were freely available.

### 3.3. Experimental design

A total of 35 male rats were assigned to seven groups of five. The study groups were assigned the following regimens: groups I, III, V and VII were treated with 0.5 mL/kg corn oil (vehicle) and groups II, IV and VI were given 200 mg/kg of vitamin E dissolve in corn oil via oral gavage. Thirty minutes later, animals were injected TCE intraperitoneally (i.p) at doses of 1000 mg/kg (groups II and III), 1500 mg/kg (groups IV and V), and 2000 mg/kg (groups VI and VII). Control rats (group I) received vehicle only. The experiment was repeated for 7 consecutive days. Twenty-four hours after the last treatment, all animals were killed with an overdose of sodium pentobarbital. Blood samples were directly taken from the left ventricle after opening the chest. Blood was collected for determination of BUN and Cr. Kidney tissues were removed and washed with normal saline, then one part of the tissue fixed and processed for light microscopy. Five histological sections, each at least 15 µm apart were taken from each tissue block and stained with hematoxylin and eosin (H&E). Other parts of the kidney tissues were collected for determination of malondialdehyde (MDA) and GSH levels.

### 3.3.1 Measurement of kidney glutathione

For the measurement of kidney GSH levels, method of Ellman was used (17). After detachment and rinsing, tissue was fragmented using surgical blade and homogenized in potassium phosphate buffer (0.1 molar, pH = 7.6) with 1/10 ratio (weigh/volume) using homogenizer at 10 000 rpm for 4 minutes. Equal volumes of trichloroacetic acid 20% and EDTA 1mM was added to homogenized tissue to precipitate protein. The mixture was shaken and incubated for 5 minutes at room temperature prior to centrifugation at 3000 rpm for 10 minutes. Then, 200 µL of supernatant was transferred to another tube to which 1.8 µL of DTNB 0.1mM (prepared in phosphate buffer 0.3M) was added and, after 5 minutes of incubation, the absorbance of yellow samples obtained was read by spectrophotometer at a wavelength of 412 nm.

Values obtained from the spectrophotometer were transformed to GSH concentration using standard curve. Blank reagent had no sample in it and blank sample lacked DNTB.

### 3.3.2. Measurement of kidney malondialdehyde

The method used is based on the reaction of MDA with thiobarbituric acid to form a colored complex, which is one type of derivatization. This assessment is adapted from the method of Buege and Aust using spectrophotometer. Calibration curve was drawn using 3, 3, 1, 1- tetraethoxypropane (TEP) that is a compound liberating MDA in acidic conditions. After detachment and rising, kidney tissue was fragmented by surgical blade and homogenized in potassium phosphate buffer (0.1 M, pH = 7.4) with 1/10 ratio (weight/volume) using homogenizer at 10 000 rpm for 4 minutes and supernatant was used for the measurement of lipid peroxidation.

The homogenized solution was centrifuged at 3000 rpm for 10 minutes. In the next step, 2 mL of thiobarbituric reactive along with 1 mL of supernatant of homogenized tissue were dispensed into capped tubes and the contents were vortexed for 10 seconds. The samples were then incubated in boiling water bath at 95-100ºC for 10 minutes and were allowed to cool at room temperature thereafter. To precipitate contaminations, samples were centrifuged at 3000 rpm for 10 minutes prior to spectrophotometry. Finally, the absorbance of pink colored samples was read at a wavelength of 532 nm and MDA concentration was calculated using standard curve (18,19).

### 3-3-4 Renal function test (BUN, Cr)

Diacetyl monoxime and Jaffe methods were used for the measurement of BUN and Cr, respectively (20,21).

### 3.4. Ethical issues

The research followed the tenets of the Declaration of Helsinki. Experimental protocol and animal care methods in the experiments were approved by the Experimental Animal Committee of Ahvaz Jundishapur University of Medical Sciences. Prior to the study, the protocol was confirmed to be in accordance with the Guidelines of Animal Ethics Committee of Ahvaz Jundishapur University of Medical Sciences.

### 3.5. Data analysis

Data of blood biochemistry and kidney were tested for homogeneity of variance and once homogeneity was confirmed, analysis of variance (ANOVA) was performed and Welch test was used otherwise. For the comparison of means to determine significant differences (*P* < 0.05), the Tukey’s test was used. In this study, five animals were assigned randomly to each group. Data was analyzed using SPSS version 23 software.

## 4- Results

### 4.1. Renal function tests

**Figure 1:**
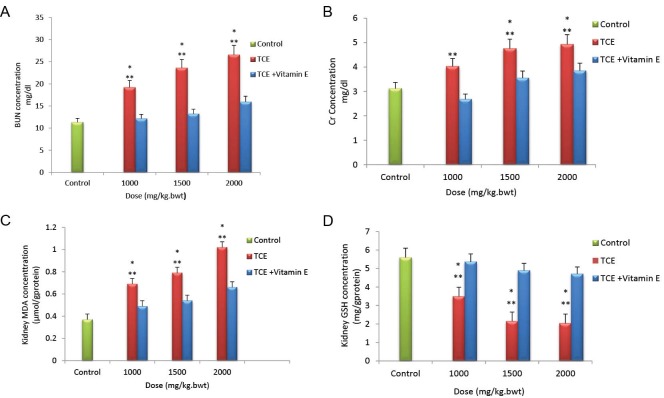
(A) Effect of vitamin E on BUN level in rats treated with TCE. (B) Effect of vitamin E on serum Cr level in rat treated with TCE. (C) Effect of vitamin E on kidney MDA content in rats treated with TCE. (D) Effects of vitamin E on kidney GSH content in rats treated with TCE.

Abbreviations: TCE, trichloroethylene; Cr, creatinine; MDA, malondialdehyde; GSH, glutathione; BUN, blood urea nitrogen. Each value represents mean ± SE of the mean.

A dose-related increase in BUN and Cr was observed in TCE-treated rats in comparison with the controls (Figures 1A and  1B). Vitamin E had no effect on blood biochemical parameters; however, pretreatment of rats with vitamin E markedly reduced all biochemical parameters in animals treated with various doses of TCE (Figures 1A and  1B).

* Significantly different from control rats (*P* < 0.05).

**Significantly different from rats pretreated with vitamin E and were given the same dose of TCE (*P* < 0.05).

### 4.2. Oxidative stress of kidney

Administration of vehicle alone did not produce detectable alteration in MDA and GSH levels. However, dose-dependent increase MDA levels and decreased GSH levels were noted in TCE-treated rats in comparison with controls (Figures 1C and 1D). Dose dependent MDA reduction and GSH elevation were observed in animals pretreated with vitamin E that had received the same dose of TCE (Figures 1C and 1D).

### 4.3. Histopathology

**Figure 2:**
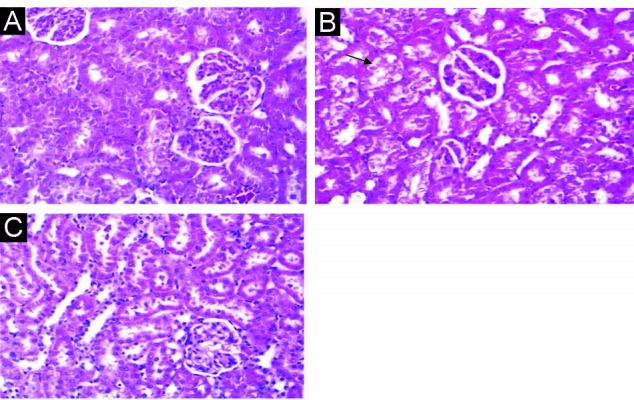
(A) Light micrograph of the control rat kidney tissue. There was no obvious injury in the kidney cells (H&E ×200). (B) .Light micrograph of rat kidney tissue treated with 2000 mg/kg TCE showing extensive injury including, dilatation of the nucleus and cellular swelling and vacuolization of renal tubular cells (arrow) (H&E ×200). (C) Light micrograph section of rat kidney tissue pretreated with 200 mg/kg vitamin E and received 2000 mg/kg TCE showing TCE induced nephrotoxicity markedly diminished in compare with non-pretreated animals which received the same dose of TCE (Figure 2B) (H&E ×200).

Administration of vehicle alone did not produced detectable injury in rat kidney and the tissue sections showed normal architecture (Figure 2A). While the TCE treated animals showed distortion in the architecture of renal morphology formation of vacuoles, tubular epithelial necrosis, dilation and loss of staining capacity (Figure 2B). The extant of injury in appeared to be a dose related manner. The most remarkable histopathological alterations were noted in animals treated with 2000 mg/kg TCE. Light microscopy revealed that renal proximal tubular cells swollen, had loss of staining capacity, and nuclei appeared to be dilated (Figure 2B). vitamin E had no effect on kidney cells, but the extent of injury markedly decreased in Vitamin E pretreatment of rats that received the same dose of TCE (Figure 2C).

## 5. Discussion

TCE is an organic solvent widely used in dry cleaning and degreasing of metal surfaces, and in the environment through TCE-polluted water sources (1,2). Therefore, the assessment of potential toxicity for individuals exposed to this substance is of great importance. The kidney is the main site for the excretion of waste products and therefore is exposed to all xenobiotics entered the body and is the target organ for TCE toxicity(1,4). BUN and Cr are sensitive biomarkers used for the evaluation of renal function and investigation of nephrotoxicity caused by toxic substances. We investigated the nephrotoxicity caused by TCE at different doses and confirmed it through increased BUN and Cr. This increase has also been reported by the oral administration of TCE in rats and mice (10,12) and by intraperitoneal injection of TCE in mice (22). Meta-analysis of epidemiological studies strongly supported the view that TCE induced adverse effects on human kidney by all route of exposure (23). We observed histopathological alteration in rat kidney treated with various doses of TCE. Histological changes was consistent with kidney biochemical alterations and further support the conclusion that TCE has potential to cause nephrotoxicity. Brüning et al found that workers exposed to TCE suffer from renal epithelial-tubular damage (24). Similarly, in vitro study showed that TCE mainly induced cytotoxicity in human and rat proximal convoluted proximal tubular cells (8). The experimental data provide evidence that metabolic pathways for TCE being qualitatively similar in humans and experimental animals including rats and mice (25,26).

The mechanism by which TCE causes nephrotoxicity involves metabolism of TCE through two irreversible pathways including cytochrome P450 and reduction of GSH conjugation. Research in toxicology has revealed that TCE associated nephrotoxicity occurs mainly through decreased GSH conjugation. Renal proximal tubule is the main site for the biotransformation of xenobiotics and cells of proximal tubule contain higher concentrations of enzymes involved in GSH conjugation. Previous studies have shown that TCE metabolites dichlorovinyl glutathione (DCVG) and dichlorovinil cysteine (DCVC) are toxic to kidney (25-30).

In the present study, the TCE-treated rats showed a significant increase in the MDA level in a dose-dependent manner. In agreement with this finding, TCE has been shown to increase the level of MDA (24,25,29,30) Our data showed vitamin E attenuated MDA level . Our finding suggested that vitamin E has potential to protect kidney against TCE induced lipid peroxidation. Acute or chronic administration of TCE increases the production of reactive oxygen species (ROS) that leads to the reduction of antioxidant level of cells and elevated oxidative stress in most tissues (29-31).

GSH is a tripeptide low molecular weight protein that plays a fundamental role in detoxifying drugs and toxins, metabolism and regulation of various pathways to maintain homeostasis. We found that exposure to TCE led to decreased GSH in a dose-dependent manner that is in agreement with other studies (12,24,29,30). Our data showed that vitamin E enhanced GSH level in TCE treated rats and protected kidney against TCE-induced oxidative stress. Zhu et al found that vitamin E prevents human epidermal keratinocytes against TCE-induced cell damage (16). These results suggest that TCE induce cytotoxicity associated with oxidative stress and vitamin E as an antioxidant agent could effectively protect cells from TCE-induced nephrotoxicity

## 6. Conclusions

The present study demonstrated that vitamin E protected the kidney against TCE-induced biochemical and histopathological alterations in rat. This renal protective effect mainly include amelioration of lipid peroxidation caused by TCE as well as elevation of GSH level.

## Conflicts of interest

The authors declared no competing interests.

## Authors’ contribution

MH provided technical assistance, collection and preparation of the manuscript. KA analyzed the data. MA designed, supervised the study and prepared the final draft of the article. All authors read and signed the final paper.

## Funding/Support

This study was supported by physiology research center and the research deputy of Ahvaz Jundishapur University of medical sciences (Grant # APRC-94-03).

## Acknowledgments

The source of data used in this paper was from master thesis of, Mojgan Heydari student of Occupational Health Engineering Department, School of Health, Ahvaz Jundishapur University of Medical Sciences, Ahvaz, Iran. Our special thanks go to Dr. B. Mohammadian for reviewing histopathologi­cal samples.
